# Patients' Characteristics Associated With Size of Ruptured and Unruptured Intracranial Aneurysms

**DOI:** 10.1002/brb3.70161

**Published:** 2024-11-28

**Authors:** Thiemo Florin Dinger, Mehdi Chihi, Meltem Gümüs, Christoph Rieß, Alejandro Nicolas Santos, Mats Leif Moskopp, Jan Rodemerk, Maximilian Schüßler, Marvin Darkwah Oppong, Yan Li, Karsten Henning Wrede, Philipp René Dammann, Ulrich Sure, Ramazan Jabbarli

**Affiliations:** ^1^ Department of Neurosurgery and Spine Surgery, and Center for Translational Neuroscience and Behavioral Science (C‐TNBS), University Hospital of Essen University of Duisburg‐Essen Essen North Rhine‐Westphalia Germany; ^2^ Department of Neurosurgery, Faculty of Medicine and University Hospital Carl Gustav Carus Technische Universität Dresden Dresden Germany; ^3^ Institute of Physiology, Medical Faculty Carl Gustav Carus Technische Universität Dresden Dresden Germany; ^4^ University Hospital Essen, Institute for Diagnostic and Interventional Radiology and Neuroradiology University of Duisburg‐Essen Essen North Rhine‐Westphalia Germany

**Keywords:** size, intracranial aneurysms, destabilizing factors

## Abstract

**Objective:**

The size of unruptured intracranial aneurysms (UIA) remains the most crucial risk factor for treatment decisions. On the other side, there is a non‐negligible portion of small ruptured IA and large stable UIA. This study aimed to identify the patients' characteristics related to IA size in the context of IA rupture status.

**Methods:**

A total of 2152 patients, with 1002 being hospitalized for an acute aneurysmal subarachnoid hemorrhage (SAH), were included from our institutional IA database. Different demographic and clinical characteristics of patients and IA were collected. IA size was the study endpoint, assessed as continuous variable in univariate and multivariable linear regression analysis, separately for ruptured (R) IA and UIA.

**Results:**

The mean IA size was 8.3 and 7.3 mm in the UIA and RIA subpopulations, respectively. Higher age (*p* = 0.003) and baseline blood urea level (*p* < 0.001) were independently associated with increasing UIA size. In contrast, location at the posterior circulation (*p* < 0.001), familiar intracranial aneurysms (*p* < 0.001), serum potassium (*p* = 0.006), and total serum protein (*p* = 0.019) were related to smaller UIA size in the multivariate analysis. For RIA, a statistically significant and independent association was detected for location (*p* = 0.019), history of gastrointestinal diseases (*p* = 0.042), and levothyroxine intake (*p* = 0.002).

**Conclusions:**

Identification of clinical characteristics related to the size of ruptured and unruptured IA allows a more differentiated view on the genesis of RIA and UIA and the value of sack size as a basis for therapeutic decision‐making. More research is needed to verify the identified risk factors.

## Graphical abstract

In a large cohort study, multiple putative size risk factors (RF) for ruptured/unruptured intracranial aneurysms could be identified. To facilitate future reporting of research and treatment, we propose hereby a comparable risk chart system, which is here used for the identified RF ([i] stabilizing/protective factors (turquoise), [ii] destabilizing factors (potentially growth‐promoting) (pink), and [iii] destabilizing factors (potentially rupture‐promoting).

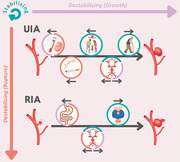

**Abbreviations**
(a)SAH: (aneurysmal) subarachnoid hemorrhage
ACA: anterior cerebral artery
ADPKD: autosomal dominant polycystic kidney disease
AHT: arterial hypertension
DSA: digital subtraction angiography
FIA: familiar intracranial aneurysms
GID: gastrointestinal diseases
HB: hemoglobin
IA: intracranial aneurysms
ICA: internal carotid artery
ISUIA: International Study of unruptured Intracranial Aneurysms
MCA: middle cerebral artery
MIA: multiple intracranial aneurysms
MRI: magnetic resonance imaging
MVA: multivariable analyses
PC: posterior circulation
RIA: ruptured intracranial aneurysms
UIA: unruptured intracranial aneurysms
UVA: using univariate analysis
WBC: white blood cell

## 1 Introduction

Counseling patients with unruptured intracranial aneurysms (UIA) can be challenging (Pagiola et al. 2020). The prevalence of UIA is about 3%–8% in the adult population, and their number is likely to increase due to the technical advances regarding magnetic resonance imaging (MRI) and the improved accessibility to cranial (vascular) imaging (Imaizumi et al. 2018). Despite a number of other clinical and radiographic risk factors, IA size is the predominant parameter to determine the rupture risk and thereby derive a treatment recommendation (Greving et al. 2014; Kamp et al. 2021; Wiebers and International Study of Unruptured Intracranial Aneurysms Investigators 2003). For example, small UIA (< 7 mm) of the anterior circulation have shown a 5‐year cumulative rupture risk of 0% in the large international multicenter natural history study of UIA (ISUIA) (Wiebers and International Study of Unruptured Intracranial Aneurysms Investigators 2003). However, this and other natural history studies of UIA inherit some bias, possibly misguiding caretakers toward the false assumption of a benign natural course for some small UIA (Guillemin 2008; Raymond et al. 2008). So, it is not surprising that an increasing number of studies showed that in up to 20%–30% of all aneurysmal subarachnoid hemorrhages (aSAH), small ruptured IA (RIA) could be identified as the source of bleeding (Dinger, Peschke et al. 2022; Ikawa et al. 2020). In other words, notwithstanding low‐risk scores, primarily based on small aneurysm sizes, a discrepancy between these scores and the RIA sizes is evident in clinical practice (Dinger, Peschke et al. 2022; Ikawa et al. 2020; Pagiola et al. 2020). So, on the one hand, there is the suspicion of underestimating the rupture risk of certain small UIA. On the other hand, autopsy studies revealed that around 10% of all detected UIA were ≥ 10 mm (Inagawa 2022; Inagawa and Hirano 1990; Iwamoto et al. 1999). Importantly, in this context, it has to be mentioned that large IA sizes are underestimated by about 3–4 mm in autopsy studies compared to studies based on digital subtraction angiography (DSA) (Inagawa 2022; McCormick and Acosta‐Rua 1970). Autopsy studies further revealed that from the age of 80, the prevalence of UIA becomes more frequent compared to RIA (Inagawa 2022). These observations support the assumption that specific clinical characteristics and/or co‐existing physiological conditions of IA carriers might act as (de‐)stabilizing factors, either promoting IA rupture at a smaller size or protecting the wall of growing IA against rupture. Such factors relativize the value of IA size in the assessment of rupture risk of UIA.

To avoid an over‐treatment of stable large IA, and the trivialization of rupture‐prone small IAs, the further elucidation of these particular conditions impacting IA size and rupture status is of paramount importance (Rautalin, Kaprio, and Korja 2020). Additionally, identifying (modifiable) factors that (de‐)stabilize the UIA wall could help modify and predict the course of UIA. Multiple studies have already identified many parameters associated with IA size/growth, like arterial hypertension (AHT), tobacco or drug consumption, female sex, prior history of SAH, IA location and morphology, and patient age (Backes et al. 2017). However, the studies comparing the value of these risk factors in the context of IA size and rupture status are lacking. Particularly, there is no study that would cover a wide range of patients' characteristics including demographic, clinical, radiographic, and baseline laboratory parameters to identify possible associations with the sack size of UIA and RIA.

So, using a large institutional IA database, our current study aimed to analyze and compare the effects of putative factors that could influence the size of UIA and RIA.

## 2 Materials and Methods

Between January 2003 and June 2016, all patients with saccular IA treated at the University Hospital of Essen, Germany, were included according to the STROBE guidelines for an observational, retrospective cohort study. Due to institutional guidelines, DSA was performed in all cases with aSAH or diagnosis/suspicion of UIA (symptomatic or asymptomatic) for IA verification. The study was approved by the Institutional Review Board (Institutional Ethical Review Committee, Medical Faculty, University of Duisburg‐Essen, registration number: 15‐6331‐BO) and registered in the German clinical trial registry (DRKS, unique identifier: DRKS00008749).

### 2.1 Definition of Study Aims

The study aimed to identify the baseline characteristics associated with IA size separately for individuals with UIA and RIA. Therefore, the patients' data were screened for socio‐demographic and radiological characteristics, preexisting medical conditions, and blood examinations.

### 2.2 Definition and Documentation of IA

IA were identified based on DSA reports of the neurocranium. Two experienced neuroradiologists at our university hospital independently reviewed the DSA images. For aSAH patients with multiple intracranial aneurysms (MIA), the RIA was allocated according to the bleeding pattern and/or the intra‐interventional findings. The exclusion criteria were: (i) missing DSA confirmation, (ii) size < 1 mm, (iii) extradural, (iv) mycotic origin, and (v) nonsaccular morphology. In the case of MIA, the RIA or the IA with the largest sack size was selected as the index IA in the subcohorts with aSAH and UIA patients, respectively (Figure S1).

### 2.3 Data Extraction

All patients' electronic charts were screened for demographic, clinical, and laboratory data. IA sizes, locations, morphologies, and numbers were extracted from DSA data. IA location was subsumed into the following groups: internal carotid artery (ICA), middle cerebral artery (MCA), anterior cerebral artery (ACA), and posterior circulation (PC, including posterior communicating, posterior cerebral, basilar, and vertebral arteries).

As previously described in detail (Dinger, Oppong et al. 2022), the patients' records were screened to extract demographic and clinical (imaging, preexisting medical conditions, ABO blood group, and blood examinations) information as summarized in Table S1. For blood examinations consisting of multiple entries, only the results at admission were included in the analysis if not stated otherwise. In addition, blood values known to be altered by aSAH and the size of its blood clot (electrolytes, blood cells, and their properties, creatine kinase, etc.) (e.g., Wartenberg et al. 2006) were excluded from the analysis of risk factors of RIA size and only assessed in an explorative manner to verify the described changes in our study. Anemia was defined for females by a hemoglobin (HB) value < 12.5 mg/dL and for males by an HB < 13.5 mg/dL.

### 2.4 Statistical Analysis

All statistical analyses were performed on SPSS (version 29.0.0.0; IBM Corporation) and OriginPro 2020 (version 9.9.0.255; OriginLab Corporation). For the statistical analysis, IA size was handled in two different ways. First, size was treated as a continuous variable for the univariate and multivariable analyses (UVA, MVA). Second, for further analysis of statistically significant variables and to check for location patterns, the IA size was dichotomized into small and large IA using the well‐accepted cut‐off of > 6 mm (Greving et al. 2014; Wiebers and International Study of Unruptured Intracranial Aneurysms Investigators 2003).

All putative predictors were checked for a significant association with IA size in UVA. Therefore, linear regression analysis was used to identify a statistically significant association. The acceptance level to commit a Type I error (*α*) was defined by < 5%. All statistically significant, putative UIA and RIA size predictors were included in the MVA. For the MVA, all missing data were handled by multiple imputations. To find statistically significant differences in location vs. size of UIA or RIA in the UVA, the Kruskal–Wallis test was used, and then the effect size was analyzed in a pairwise method with post hoc analysis (Dunn's test). The chi‐square test was used to compare small vs. large (dichotomized) IA at the different locations.

## 3 Results

For the final analysis, 2152 patients/IA were included, with a total of 1002 aSAH/RIA cases (Figure S1).

**FIGURE 1:**
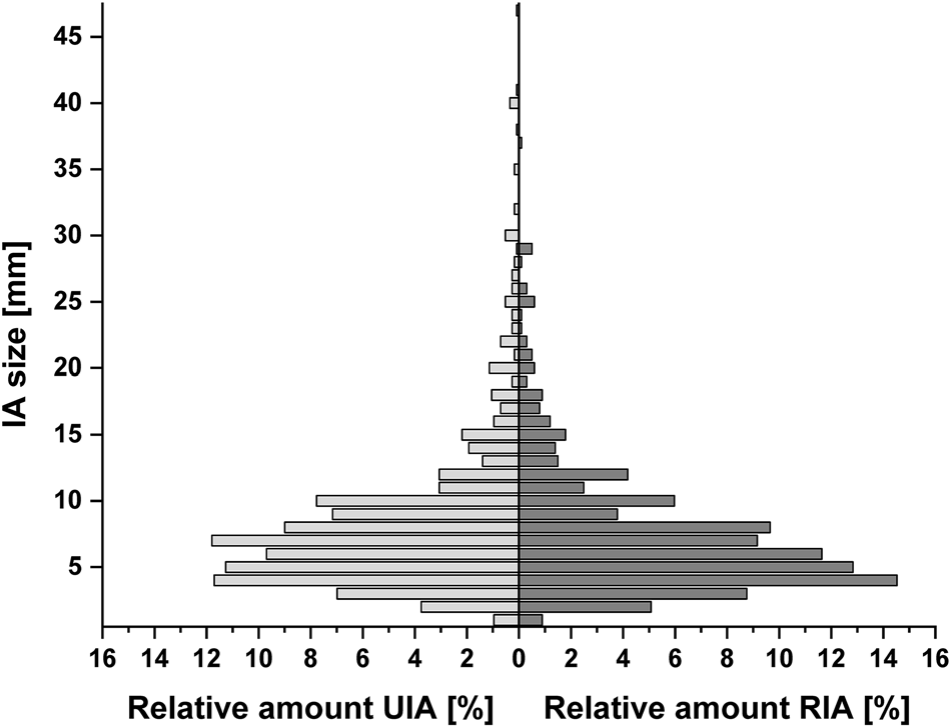
Pyramid plot depicting the relative size distribution in the UIA subpopulation (left) and the RIA subpopulation (right).

In the subpopulation of UIA, the mean age was 55.1 years, with 71.0% being female. Regarding the RIA subpopulation, the mean age was 54.4 years, with 67.7% female patients (Table S1). The mean IA size was 8.3 mm (interquartile range [IQR]: 5–7 mm) and 7.4 mm (IQR: 4–9 mm) for UIA and RIA, respectively (Table S1). In Figure 1, the size distribution of the UIA and RIA is depicted.

### 3.1 Unruptured Intracranial Aneurysms

#### 3.1.1 Size of UIA—Demographic Aspects

Increasing UIA size correlated statistically significantly with increasing age (*p* < 0.001) in the UVA (Table 1). Female sex was not significantly associated with UIA size (*p* = 0.561, Table 1). Also, the ethnicity did not show a statistically significant association with increasing UIA size (*p* = 0.438, Table 1).

**TABLE 1:** Univariate linear regression analysis of putative demographic and clinical risk/protective factors regarding the size of unruptured intracranial aneurysms.

Parameter class	*p*‐value	95% CI	Coefficient
Putative RF			
**Demographic**			
Age (years)	< 0.001	>0.02 to 0.07	>0.047
Female	0.561	−0.52 to 0.96	0.220
Ethnicity (non‐Caucasian)	0.438	−2.09 to 0.90	−0.591
**Imaging (findings)**			
MIA	0.535	−0.49 to 0.95	0.227
IA location	< 0.001	—	—
Location PC^a^	< 0.001	0.69 to 2.40	1.544
**Preexisting medical conditions**			
Adiposity	0.065	−0.09 to 2.77	1.344
ADPKD	0.655	−2.60 to 1.63	−0.482
AHT	0.346	−0.36 to 1.03	0.332
Alcohol abuse	0.908	−3.38 to 3.00	−0.188
Anemia	0.065	−2.21 to 0.07	−1.072
Cardiac diseases	0.811	−0.87 to 1.12	0.121
Chronic inflammation	0.824	−1.77 to 1.41	−0.181
Diabetes	0.978	−1.11 to 1.14	0.016
Drug abuse	0.259	−1.36 to 5.02	1.835
Dyslipidemia	0.302	−1.25 to 0.39	−0.430
FIA	0.037	−3.23 to −0.10	−1.665
Gastrointestinal diseases	0.153	−0.31 to 1.97	0.831
Gynecologic diseases	0.344	−4.31 to 1.51	−1.404
Hepatic diseases	0.349	−2.90 to 1.03	−0.937
Hyperthyroidism	0.871	−2.37 to 2.80	0.214
Hyperuricaemia	0.517	−3.35 to 1.69	−0.831
Hypothyroidism	0.556	−1.09 to 0.59	−0.252
Musculoskeletal diseases	0.151	−2.08 to 0.32	−0.881
Oncologic disease	0.922	−1.10 to 0.99	−0.052
Peripheral arterial diseases	0.365	−1.33 to 0.49	−0.419
Pulmonary diseases	0.439	−1.72 to 0.75	−0.486
Renal diseases	0.347	−0.62 to 1.75	0.566
Smoker	0.046	−1.66 to −0.02	−0.839
**Blood groups**			
O	0.498	−0.47 to 1.02	0.262
A	0.173	−1.26 to 0.23	−0.514
B	0.268	−0.50 to 1.81	0.655
AB	0.982	−1.71 to 1.67	−0.020
**Blood examination**			
ALT [U/L]	0.913	−0.02 to 0.02	0.001
AST [U/L]	0.976	−0.03 to 0.03	0.000
(t)BIL [mg/dL]	0.555	−1.70 to 0.92	−0.394
Calcium [mmol/L]	0.363	−3.69 to 1.35	−1.168
Chloride [mmol/L]	0.833	−0.11 to 0.08	−0.010
CK [U/L]	0.726	−0.01 to 0.00	−0.001
Creatinine [mg/dL]	0.074	−0.07 to 1.43	0.680
CRP [mg/dL]	0.296	−0.15 to 0.05	−0.051
γ‐GT [U/L]	0.440	−0.01 to 0.00	−0.002
HB [mg/dL]	0.626	−0.20 to 0.33	0.066
HCT [%]	0.419	−0.06 to 0.13	0.039
LDH [U/L]	0.527	−0.01 to 0.01	−0.002
MCH [pg]	0.756	−0.21 to 0.16	−0.029
MCV [fL]	0.863	−0.06 to 0.08	−0.006
Phosphate [mmol/L]	0.569	−0.40 to 0.72	0.163
PLT [/nL]	0.829	0.00 to 0.01	0.001
Potassium [mmol/L]	0.006	−2.21 to −0.38	−1.292
RBC [/pL]	0.634	−0.60 to 0.99	0.193
Sodium [mmol/L]	0.019	0.03 to 0.28	0.151
TP [g/dL]	0.009	−1.36 to −0.20	−0.779
Urea [mg/dL]	0.002	0.04 to 0.15	0.092
WBC [/nL]	0.774	−0.17 to 0.23	−0.022
**Prescribed drugs**			
β‐blocker	0.960	−0.75 to 0.72	−0.019
ACE‐inhibitors	0.333	−0.39 to 1.15	0.381
ASA	0.595	−0.73 to 1.28	0.272
AT1‐antagonists	0.246	−1.52 to 0.39	−0.566
Calcium‐antagonists	0.328	−0.43 to 1.29	0.430
Levothyroxine	0.530	−1.23 to 0.63	−0.297
Statins	0.196	−1.44 to 0.30	−0.572

*Note*: The Kruskal–Wallis test was used to analyze the variable “IA location.” Statistically significant factors are highlighted in grey.

Abbreviations: ACE, angiotensin‐converting enzyme; ADPKD, autosomal‐dominant polycystic kidney disease; AHT, arterial hypertension; ALT, alanine transaminase; ASA, acetylsalicylic acid; AST, aspartate transaminase; AT1, angiotensin receptor 1; CK, creatine kinase; CRP, C‐reactive protein; FIA, familial intracranial aneurysms; HB, hemoglobin; HCT, hematocrit; LDH, lactate dehydrogenase; MCH, mean corpuscular/cellular hemoglobin; MCV, mean corpuscular volume; MIA, multiple intracranial aneurysms; PLT, platelet count; RBC, red blood cell count; (t)BIL, total bilirubin; TP, total protein; WBC, white blood cell count; γ‐GT, γ‐glutamyltransferase.

^a^ Dichotomized variable compared to Table S1.

#### 3.1.2 Size of UIA—Imaging Results

In the next step, (putative) IA (size) risk/protective imaging factors were analyzed for a correlation with increasing UIA size. The presence of MIA did not show a significant association with UIA size (*p* = 0.535, Table 1). Furthermore, the UIA location significantly correlated with UIA size (*p* < 0.001, Table 1). Using the Kruskal–Wallis post hoc analysis, a statistically significant difference could be revealed for UIA at the PC vs. MCA and PC vs. ACA (*p* < 0.001; Figure S2). In whole, the location at PC (vs. any other location) showed a statistically significant regression with UIA size (*p* < 0.001, Table 1).

**FIGURE 2:**
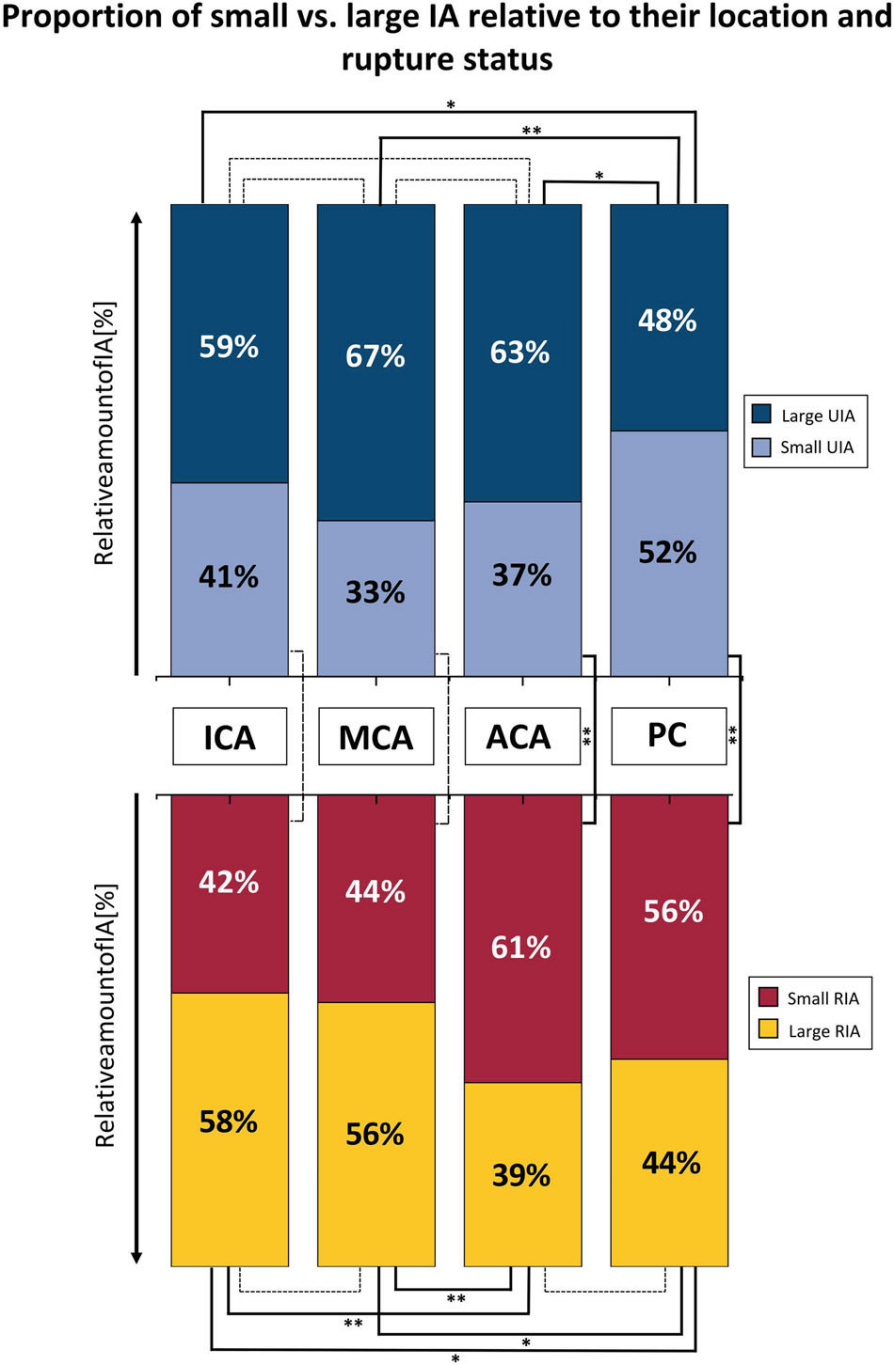
(Upper part) Stacked column plot of small UIA (< 7 mm; light blue) and large UIA (≥ 7 mm; dark blue) for each location (ICA, MCA, ACA, and PC). The lower part shows an equivalent stacked column plot for small RIA (red) vs. large RIA (yellow). The lines indicate if there is a statistically significant difference by comparing the connected groups (dotted line: *p* > 0.05; solid line with *p < 0.05; solid line with **p < 0.001).

For the subsequent analysis, IA size was dichotomized into small and large UIA (cut‐off defined by > 6 mm). Small vs. large UIA proportions at the PC (52% vs. 48%) differed significantly from all other locations (Figure 2, upper part).

#### 3.1.3 Size of UIA—Preexisting Medical Conditions

Summarized in Table 1, the risk factors ADPKD, AHT, alcohol abuse, diabetes, and renal diseases did not show a statistically significant association with the size of UIA in our study (*p* = 0.665, *p* = 0.346, *p* = 0.908, *p* = 0.978, and *p* = 0.347, respectively). In contrast, familiar intracranial aneurysms (FIA) showed a statistically significant correlation with UIA size (*p* = 0.037, Table 1). Also, tobacco consumption correlated with UIA size (*p* = 0.046, Table 1). None of the analyzed medications correlated significantly with UIA size (Table 1).

#### 3.1.4 Size of UIA—Blood Examination

The systemic inflammation markers, CRP, and white blood cell (WBC) count did not correlate significantly with UIA size (*p* = 0.296 and *p* = 0.774, respectively; Table 1). Analyzing the electrolyte levels in UIA patients, a statistically significant regression was found for potassium (negative regression, *p* = 0.006) and sodium (positive regression, *p* = 0.019) (Table 1). Also, the urea level correlated significantly with the UIA size (*p* = 0.002, Table 1). The additional functional kidney marker, creatinine, did not correlate with UIA size (*p* = 0.074). In contrast, the total protein level was statistically significantly associated with UIA size (*p* = 0.009; Table 1).

#### 3.1.5 Size of UIA—Multivariable Analysis

In the final MVA, age correlated significantly with UIA size (*p* = 0.003, Table 2). Other UIA size risk factors that remained statistically significant in the MVA were IA location (PC) and FIA (both *p* < 0.001; Table 2). In contrast, smoking did not remain statistically significant in MVA (*p* = 0.112; Table 2). Regarding the blood examination, potassium, total protein, and urea levels remained statistically significant in MVA for UIA size (*p* = 0.006, *p* = 0.019, and *p* < 0.001, respectively; Table 2).

**TABLE 2:** Multivariable linear regression model of (potential) risk/protective factors for the size of unruptured intracranial aneurysms.

Parameter class	*p*‐value	95% CI	Coefficient
Putative RF			
**Demographics**			
Age	0.003	0.01 to 0.07	2.946
**Imaging findings**			
PC^a^	< 0.001	0.63 to 2.33	3.425
**Preexisting medical conditions**			
FIA	< 0.001	−3.09 to −0.01	−1.962
Smoker	0.122	−1.47 to 0.17	−1.548
**Blood examination**			
**(Separate MVA)**			
Potassium [mmol/L]	0.006	−2.27 to −0.40	−2.807
Sodium [mmol/L]	0.057	0.00 to 0.26	1.930
TP [g/dL]	0.019	−1.29 to −0.12	−2.369
Urea [mg/dL]	< 0.001	0.04 to 0.15	3.339

*Note*: Statistically significant risk/protective factors (RF), the p‐value, the 95% confidence interval (CI), and the regression coefficient are highlighted in grey.

Abbreviations: FIA, familial intracranial aneurysms; PC, posterior circulation; TP, total protein.

^a^ Dichotomized variable compared to Table_Patients‐Characteristics.

### 3.2 Ruptured Intracranial Aneurysms

#### 3.2.1 Size of RIA—Demographic Aspects

Increasing size of RIA did not significantly correlate with increasing age (*p* = 0.169) in the UVA (Table 3). Additionally, female sex and ethnicity did not significantly correlate with RIA size (*p* = 0.690 and *p* = 0.430, respectively; Table 3).

**TABLE 3:** Univariate linear regression analysis of putative demographic and clinical risk/protective factors regarding the size of ruptured intracranial aneurysms.

Parameter class	*p*‐value	95% CI	Coefficient
Putative RF			
Demographic			
Age (years)	0.169	−0.04 to 0.01	−0.015
Female	0.690	−0.50 to 0.76	−0.129
Ethnicity (non‐Caucasian)	0.430	−0.84 to 1.97	−0.564
**Imaging (findings)**			
Multiplicity	0.050	0.00 to 1.29	−0.643
IA location	< 0.001	—	—
**Preexisting medical conditions**			
Adiposity	0.576	−0.69 to 1.25	0.276
ADPKD	0.463	−4.33 to 1.97	−1.180
AHT	0.102	−1.17 to 0.11	−0.532
Alcohol abuse	0.626	−0.84 to 1.40	0.279
Anemia	0.072	−1.52 to 0.07	−0.729
Cardiac diseases	0.721	−0.61 to 0.89	0.136
Chronic inflammation	0.620	−1.30 to 0.78	−0.263
Diabetes	0.282	−1.58 to 0.46	−0.558
Drug abuse	0.459	−1.23 to 2.73	0.747
Dyslipidemia	0.268	−1.37 to 0.38	−0.496
FIA	0.656	−2.11 to 3.35	−0.620
Gastrointestinal diseases	0.031	−1.68 to −0.08	−0.880
Gynecologic diseases	0.372	−1.92 to 0.72	−0.600
Hepatic diseases	0.736	−1.65 to 1.16	−0.241
Hyperuricaemia	0.654	−2.21 to 1.39	−0.412
Musculoskeletal diseases	0.524	−0.57 to 1.12	0.274
Oncologic disease	0.366	−1.47 to 0.54	−0.464
Peripheral arterial diseases	0.995	−0.92 to 0.92	0.003
Pulmonary diseases	0.383	−0.51 to 1.32	0.407
Renal diseases	0.290	−1.26 to 0.38	−0.443
Smoker	0.482	−0.42 to 0.88	0.232
**Blood groups**			
O	0.366	−1.04 to 0.38	−0.327
A	0.382	−0.39 to 1.01	−0.312
B	0.288	−0.51 to 1.72	0.604
AB	0.115	−3.06 to 0.33	−1.362
**Blood examination**			
ALT [U/L]	0.558	−0.01 to 0.01	0.003
AST [U/L]	0.203	0.00 to 0.02	0.006
(t)BIL[mg/dL]	0.348	−1.41 to 0.50	−0.455
(t)BIL (> 1.2) [mg/dL]	0.672	−1.16 to 1.80	0.320
Calcium [mmol/L]	0.014	−5.00 to ‐0.56	−2.778
Chloride [mmol/L]	0.089	−0.01 to 0.11	0.053
CK [U/L]	0.091	0.00 to 0.00	0.000
Creatinine [mg/dL]	0.187	−1.75 to 0.34	−0.701
CRP [mg/dL]	0.717	−0.09 to 0.06	−0.014
γ‐GT [U/L]	0.449	−0.01 to 0.00	−0.001
HB [mg/dL]	0.293	−0.09 to 0.30	0.105
HCT [%]	0.183	−0.02 to 0.12	0.050
LDH [U/L]	0.794	0.00 to 0.01	0.001
MCH [pg]	0.971	−0.14 to 0.14	0.003
MCV [fL]	0.950	−0.06 to 0.06	0.002
Phosphate [mmol/L]	0.617	−0.56 to 0.33	−0.114
PLT [/nL]	0.533	0.00 to 0.01	0.002
Potassium [mmol/L]	0.256	−1.08 to 0.29	−0.396
RBC [/pL]	0.190	−0.21 to 1.08	0.431
Sodium [mmol/L]	0.457	−0.13 to 0.06	−0.037
TP [g/dL]	0.213	−0.66 to 0.15	−0.258
Urea [mg/dL]	0.157	−0.08 to 0.01	−0.034
WBC [/nL]	0.010	0.02 to 0.17	0.096
**Prescribed drugs**			
β‐blocker	0.759	−0.70 to 0.95	0.129
ACE‐inhibitors	0.605	−1.00 to 0.58	−0.209
ASA	0.428	−0.72 to 1.70	−0.490
AT1‐antagonists	0.874	−1.19 to 1.40	0.105
Calcium‐antagonists	0.937	−1.05 to 0.97	−0.041
Levothyroxine	0.005	0.43 to 2.39	1.411
Statins	0.973	−1.03 to 0.99	−0.018

*Note*: The Kruskal–Wallis test was used to analyze the variable “IA location.” Putative risk/protective factors with a statistically significant correlation are highlighted in grey.

Abbreviations: ACE, angiotensin‐converting enzyme; ADPKD, autosomal‐dominant polycystic kidney disease; AHT, arterial hypertension; ALT, alanine transaminase; ASA, acetylsalicylic acid; AST, aspartate transaminase; AT1, angiotensin receptor 1; CK, creatine kinase; CRP, c‐reactive protein; FIA, familial intracranial aneurysms; HB, hemoglobin; HCT, hematocrit; LDH, lactate dehydrogenase; MCH, mean corpuscular/cellular hemoglobin; MCV, mean corpuscular volume; PLT, platelet count; RBC, red blood cell count; (t)BIL, total bilirubin; TP, total protein; WBC, white blood cell count; γ‐GT, γ‐glutamyltransferase.

#### 3.2.2 Size of RIA—Imaging Results

RIA location significantly correlated with the sack size (*p* < 0.001, Table 3). The subsequent Kruskal–Wallis post hoc analysis demonstrated significant differences between the following pairs: ACA vs. MCA (*p* < 0.001) and ACA vs. ICA (*p* < 0.001) (Figure S2), with RIA located at the ACA being significantly smaller.

To further analyze the association of RIA size and location, size was dichotomized similarly to what was previously done for UIA (Figure 2, lower part). Two groups could be identified. First, RIA located at the ICA and MCA had a higher proportion of large than small RIA (58% and 56% of large IA, respectively, Figure 2). Second, RIA located at the ACA and PC had more “small IA” than “large IA” (61% and 56% of small RIA, respectively, Figure 2). All comparisons between the locations of the first group (ICA & MCA) and the second group (ACA & PC) showed a statistically significant difference (Figure 2, lower part).

In an additional analysis regarding location, the proportions of small and large IA were compared between RIA and UIA separately for each location (Figure 2, upper vs. lower part). A statistically significant difference could be observed for the ACA and PC locations. The proportion of small IA was significantly higher regarding RIA cases than UIA cases for both locations.

#### 3.2.3 Size of RIA—Preexisting Medical Conditions

ADPDK, AHT, FIA, and tobacco consumption did not correlate with RIA size (*p* = 0.463, *p* = 0.102, *p* = 0.656, and *p* = 0.482, respectively; Table 3). A statistically significant regression with the size of RIA could be detected for gastrointestinal diseases (GID) (*p* = 0.031) (Table 3). Additionally, regarding RIA size, levothyroxine intake was statistically significant (*p* = 0.005; Table 3).

#### 3.2.4 Size of RIA—Multivariable Analysis

In the MVA, IA location remained significant (*p* = 0.019) (Table 4). Also, GID and levothyroxine intake were statistically significant in MVA (*p* = 0.042 and *p* = 0.002, respectively) (Table 4).

**TABLE 4:** Multivariable linear regression model of (putative) risk/protective factors for the size of ruptured intracranial aneurysms.

Parameter class	*p*‐value	95% CI	Coefficient
**Putative RF**			
**Imaging findings**			
IA location	0.019	−0.44 to −0.04	−0.241
**Preexisting medical conditions**			
Gastrointestinal diseases	0.042	−1.62 to −0.03	−0.825
**Prescribed drugs**			
Levothyroxine	0.002	0.56 to 2.52	1.538

*Note*: Statistically significant risk/protective factors (RF), the *p*‐value, 95% confidence interval (CI), and regression coefficient are highlighted in gray.

#### 3.2.5 Combined Analysis of UIA and RIA Risk Factors

**FIGURE 3:**
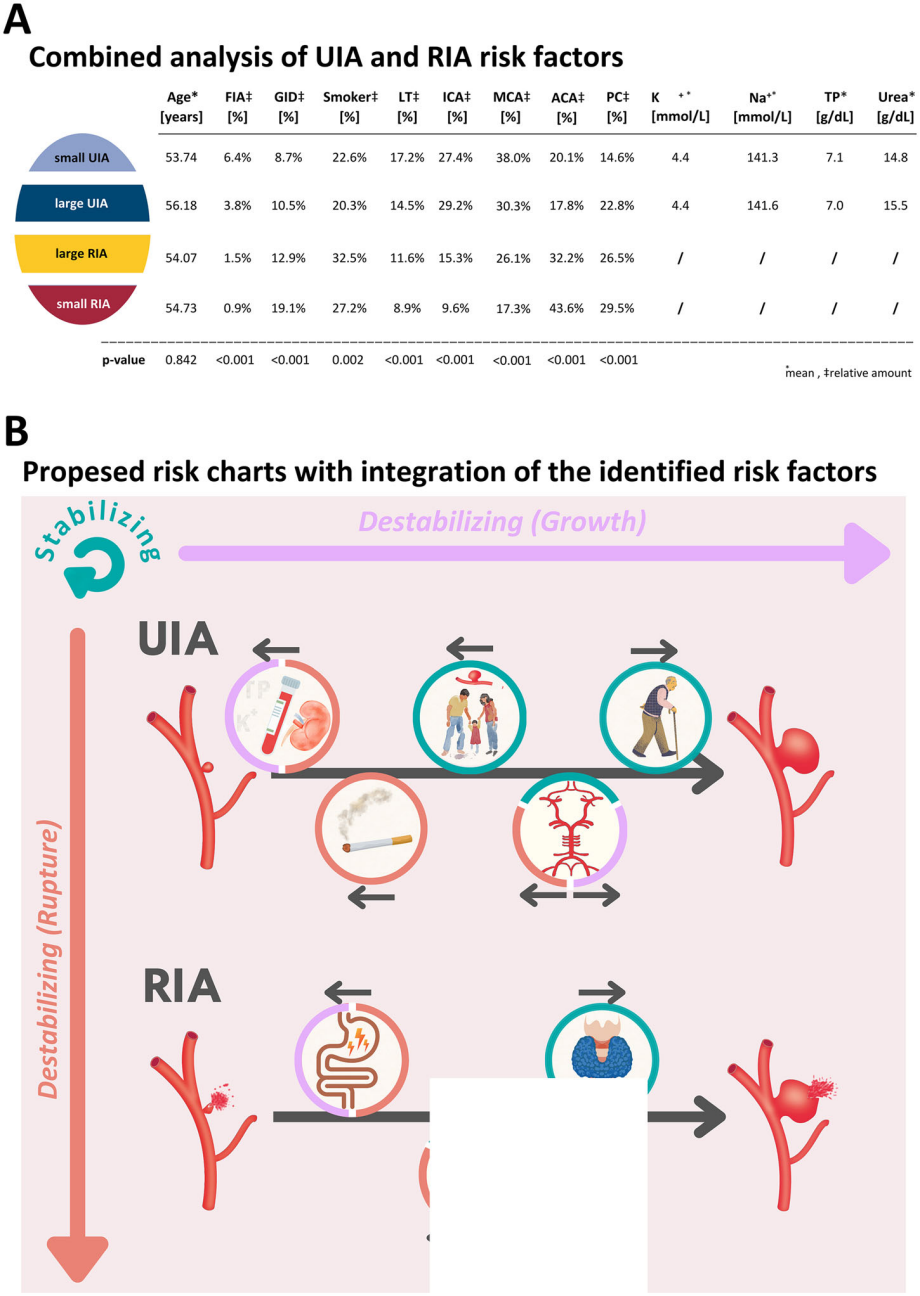
(A) The upper table shows a combined analysis of UIA and RIA after size was dichotomized using a cut‐off of 7 mm. The table shows the relative amounts or mean values of the risk factors that were identified by the UVA or MVA for each of the four subgroups: small UIA (light blue), large UIA (dark blue), large RIA (yellow), and small RIA (red). Linear regression analysis was used to check for statistical significance (*p* < 0.05). (B) This schematic illustration summarizes the results after their integration into the current scientific context. The identified risk factors, depicted by symbols, were placed according to the study's results (association with UIA upper line) or RIA (lower line) with a small arrow above their symbol to indicate the direction of their correlation with IA size. The circle color around the symbols demonstrates the category to which the factors were classified. As explained in detail in the discussion, parameters could be classified into different groups: (i) stabilizing/protective factors (turquoise), (ii) destabilizing factors (potentially growth‐promoting) (pink), and (iii) destabilizing factors (potentially rupture‐promoting). Hopefully, in the future, risk factors can be directly visualized in a comparable risk chart, depicting stabilizing and destabilizing properties of factors and helping caregivers and patients understand each patient's individual situation and thereby tailor their treatments.

All identified risk factors of UIA and RIA size were further analyzed in a combined manner by defining four subcategories: (i) small UIA, (ii) large UIA, (iii) large RIA, and (iv) small RIA. Except for age (*p* = 0.842), all other identified UIA and RIA risk factors showed a statistically significant regression with the four risk groups (all *p* < 0.001; except smoking *p* = 0.002; Figure 3A). Although the relative number of FIA patients decreases with increasing subcategories, the effect was contrary for smokers and patients suffering from GID (Figure 3A). Also, these two parameters demonstrated a correlation with the rupture status and/or size (destabilizing factors); they do also differ. Whereas GID had a slight relative increase from small UIA to large UIA of 1.8%, there is a decrease of 2.3% regarding tobacco consumption. Comparing the amounts of small RIA with small UIA and large RIA with large UIA, an increase of 10.4% and 2.4%, respectively, for GID and 4.6% and 12.2%, respectively, for smokers could be observed (Figure 3A). Levothyroxine intake, location at the ICA, and MCA revealed a decreasing relative number of patients comparing the subcategories “small UIA” to “small RIA” (Figure 3A). Locations with statistically significant opposite associations regarding the subcategories were ACA and PC, with a comparable trend like tobacco consumption mostly influencing the rupture status of the IA. The highest percentages of small RIA were located at the ACA (43.6%) and PC (29.5%). In contrast, the lowest percentages regarding these two locations were detected in the subgroup of small UIA (20.1% and 14.6%, respectively; Figure 3A).

## 4 Discussion

In this study, many known and suspected risk factors of IA were checked for an association with the size of UIA and RIA at the time of diagnosis. Specifically, age, IA location, FIA, smoking, specific blood results, GID, and levothyroxine intake were associated with IA size and rupture status.

The known risk factor of tobacco consumption showed a significant regression with a small UIA size at diagnosis compared to non‐smokers in the UVA. However, this factor did not remain significant in the MVA. From previous studies, we know that smoking is a substantial risk factor for IA rupture (Can et al. 2017). This implies that in smokers, IA might tend to an earlier rupture at smaller sack size without further growth to a larger IA.

The location of IA is another well‐established factor in the risk assessment of IA. The strongest association in our study was found for IA located at the PC and ACA. The proportions of small vs. large aneurysms for these locations in UIA vs. RIA showed completely different patterns. Meanwhile, in the UIA group, the amount of small IA is 37% and 52% for ACA and PC, respectively, and these relative amounts of small IA changed to 61% and 56% regarding RIA. The literature has described that IA located at the ACA and PC rupture at a smaller size than IA of the ICA and MCA (Dinger, Peschke et al. 2022; Ikawa et al. 2020; Wiebers and International Study of Unruptured Intracranial Aneurysms Investigators 2003). Our results together with the previously mentioned references furthermore point out the risk of a small IA of the ACA. This location should be assessed similarly to UIA of the PC instead of summarizing ICA, MCA, and ACA UIA as IA of the anterior circulation as done by the ISUIA trial (Wiebers and International Study of Unruptured Intracranial Aneurysms Investigators 2003).

Even though a wealth of publications supports the higher risk of rupture of small ACA and PC IA, there are contradictory data, which should be mentioned here for completeness. As an example, van der Kamp et al. (2021) revealed in a work on growing UIA that IA of the MCA presented with the highest rupture rate compared to other locations. However, it appears conclusive that, due to the different levels of wall shear stress and other changing rheological factors at different locations, the wall structure and the size dynamics of an UIA differ (Dinger, Oppong et al. 2022; Soldozy et al. 2019; Turjman, Turjman, and Edelman 2014).

The other known identified risk factors are FIA and age. FIA was a size‐associated factor in our study that remained significant even in the MVA. We suspect a screening effect of FIA patients that could have led to an overrepresentation of FIA patients with a small UIA. Additionally, FIA is also an accepted rupture risk factor, which could also explain the regression with small UIA size, comparable to tobacco consumption (Mensing et al. 2019). Age also remained significant in the MVA with increasing UIA size. Previous studies have already described an association between IA size and increasing age (Burns et al. 2009). This is most likely caused by a cumulative effect of other size‐affecting factors over the lifetime. Moreover, the presence of an association between age and UIA size but not the size of RIA underlines the assumption on the different nature and genesis of UIA and RIA (K. Kataoka et al. 2000). So, the prevalence of the wall stabilizing over the rupture‐driving factors explains the increasing size of IA with increasing age in the case of a stable UIA. In contrast, specific acute conditions or the extent of exposure to known IA risk factors (i.e., tobacco consumption or blood pressure levels) might lead to the rupture of unstable IA regardless of patients' age (Can et al. 2017; Ikawa et al. 2020).

A recent observation in the pathophysiology of IA has been described for the thyroid‐regulated metabolism (Dinger, Oppong et al. 2022; H. Park et al. 2023). H. Park et al. (2023) found an association between a long‐term thyroid hormone replacement for hypothyroidism and a reduced IA rupture risk in a large Korean epidemiologic study. The authors additionally found that the RIA of patients with levothyroxine intake was statistically significantly larger than in patients who did not take thyroid hormone replacement. A protective role of this medical condition is hypothesized, but the underlying pathophysiology is unknown. As described previously, we hypothesized a reduced CNS metabolism to cause this effect (Dinger, Oppong et al. 2022). Thus, a commonly observed inadequate levothyroxine administration (e.g., undertreatment) (Okosieme et al. 2011)/the altered CNS metabolism (Głombik et al. 2020) could also have an effect on IA size and rupture risk.

Renal insufficiency is a known IA risk factor that did not show a statistical association with UIA or RIA size in our study. However, an increased urea blood level remained significant in the MVA for UIA size. We hypothesized that a subclinical/nondiagnosed renal insufficiency might be associated with UIA size. H. Kataoka et al. (2022) found a statistically significant increase in serum urea levels in ADPKD patients with IA compared to those without IA. This observation indicates that subclinical renal dysfunction with its complications, such as blood pressure dysregulations, may be present in our study patients with larger UIA/RIA. Additionally, the total blood protein level may alter the blood pressure/rheology and influence IA size, as previously described in detail (Dinger, Oppong et al. 2022).

Less is known regarding the serum potassium level that we found to be statistically significantly associated with UIA size. A study revealed, in an in vivo rat model, a potassium‐related antagonizing effect on NaCl‐induced AHT, which could be beneficial for IA patients (Goto, Tobian, and Iwai 1981). Additionally, a potassium‐mediated vasodilatation has been published for the CNS with a reduced/normalized wall shear stress (Bekar and Nedergaard 2013).

Last, little is known about the putative link between IA and GID, but it could be shown that depending on the gut and oral microbiota, there was either a protective or potential negative influence on the appearance of hemorrhagic stroke and IA pathophysiology in general (Joerger et al. 2023; Shen et al. 2023; Zhang et al. 2024). Despite the limited knowledge about the direct link between GID and IA, a substantial body of research supports the hypothesis of a strong influence/promotion of GID on neurological diseases, such as migraine, epilepsy, Parkinson's disease, cerebrovascular diseases, or multiple sclerosis (Barcellos et al. 2006; Casella et al. 2013; J. W. Park et al. 2013; Skeen 2002; Zeng et al. 2022). Studies that tried to unravel the underlying pathophysiology could show a pathophysiologically altered (i) intestinal barrier, (ii) brain‐gut‐axis, and (iii) gut microbiome in these conditions (Camara‐Lemarroy et al. 2018). All these changes promote increased systematic and central nervous inflammation, thrombosis, and malnutrition syndromes with accompanying CNS symptoms (Camara‐Lemarroy et al. 2018). Inevitably, it is evident that these circumstances also favor the pathophysiology of IA, as a critical step in this is inflammation of the vessel wall (Frösen et al. 2012; Turjman, Turjman, and Edelman 2014).

In a final step, we tried to improve the validity of the risk factors by a combined analysis of UIA and RIA (Figure 3A). This analysis offers the opportunity to classify the parameters into three risk groups: (i) stabilizing, (ii) destabilizing (growth‐ or rupture‐associated), and (iii) strongly destabilizing (growth‐ and rupture‐associated). Demonstrating reduced amounts of ruptured and/or large IA, IA location at the ICA or MCA, and levothyroxine intake could be classified as stabilizing factors (Figure 3A,B), whereas the results regarding FIA status should be rather interpreted according to the screening effect of this association. As GID and IA location at PC correlated with size and rupture status in the combined analysis, they should be considered as strongly destabilizing factors. Finally, smoking and IA location at the ACA were rupture‐associated destabilizing factors. By using the proposed classification of risk factors in the future, we hope that the risk assessment of UIA will be more structured and easier to understand for caregivers and patients. Ultimately, the goal of further research using a standardized risk nomenclature for UIA will be to identify “stable” UIA and “unstable” (small) UIA or stabilizing/destabilizing modifiers, as this could spare patients from potentially harmful procedures or SAH and even allow a medical prevention of rupture. Additionally, individual IA risk stratification could give more options to treat multimorbid patients with “large” UIA conservatively (Liu et al. 2022).

### 4.1 Limitations

The main limitation of this study is its monocentric, retrospective design. The completeness and reliability of data are thereby limited. The study also carries the risk of selection/center bias. Likely, some patients with small UIA have not been referred to our university hospital by general practitioners, outpatient neurologists, neurosurgeons, and radiologists. Additionally, the cross‐sectional design of this study did not allow to investigate IA growth and changes in risk factors over time (e.g., blood test). Further multicentric, prospective natural course studies are needed to validate our results.

## 5 Conclusions

Size of UIA is still considered the most crucial risk factor for rupture and making treatment decisions. By identifying known and new factors concerning IA size, a further step has been taken towards a more differentiated view of IA size as a basis for decision‐making. Depending on the IA's location and the patient's age and general medical condition (e.g., thyroid hormone replacement, GID, tobacco consumption), treatment options for IA of the same size could differ. More research is needed to verify the identified risk factors. Still, our results might help to potentially use the modifiable factors and serum markers as therapeutic and diagnostic options in the future.

## Author Contributions

**Thiemo Florin Dinger**: conceptualization, investigation, funding acquisition, writing–original draft, methodology, visualization, software, formal analysis, data curation, resources. **Mehdi Chihi**: investigation. **Meltem Gümüs**: investigation. **Christoph Rieß**: investigation. **Alejandro Nicolas Santos**: investigation, writing–review and editing. **Mats Leif Moskopp**: writing–review and editing, formal analysis. **Jan Rodemerk**: writing–review and editing. **Maximilian Schüßler**: investigation, writing–review and editing. **Marvin Darkwah Oppong**: investigation, writing–review and editing, conceptualization, supervision. **Yan Li**: supervision, investigation. **Karsten Henning Wrede**: supervision, investigation. **Philipp René Dammann**: supervision. **Ulrich Sure**: supervision, validation. **Ramazan Jabbarli**: conceptualization, investigation, funding acquisition, writing–original draft, writing–review and editing, validation, formal analysis, project administration, supervision, resources.

## Conflicts of Interest

Dr. Wrede received personal fees from Biogen for expert opinion on aneurysms and vestibular schwannomas. The other authors declare no conflicts of interest.

### Peer Review

The peer review history for this article is available at https://publons.com/publon/10.1002/brb3.70161.

## Supporting information



Supporting Information.

## Data Availability

The data that support the findings of this study are available on request from the corresponding author. The data are not publicly available due to privacy or ethical restrictions.
