# Supplementary material for: Patients' Characteristics Associated With Size of Ruptured and Unruptured Intracranial Aneurysms
Source: Brain Behav. 2024 Nov 28;14(11):e70161. doi: 10.1002/brb3.70161 (PMC11603431; doi:10.1002/brb3.70161)
Supplement: Supplementary file 1 — Supporting Information. [file BRB3-14-e70161-s001.pdf]

Figure S1

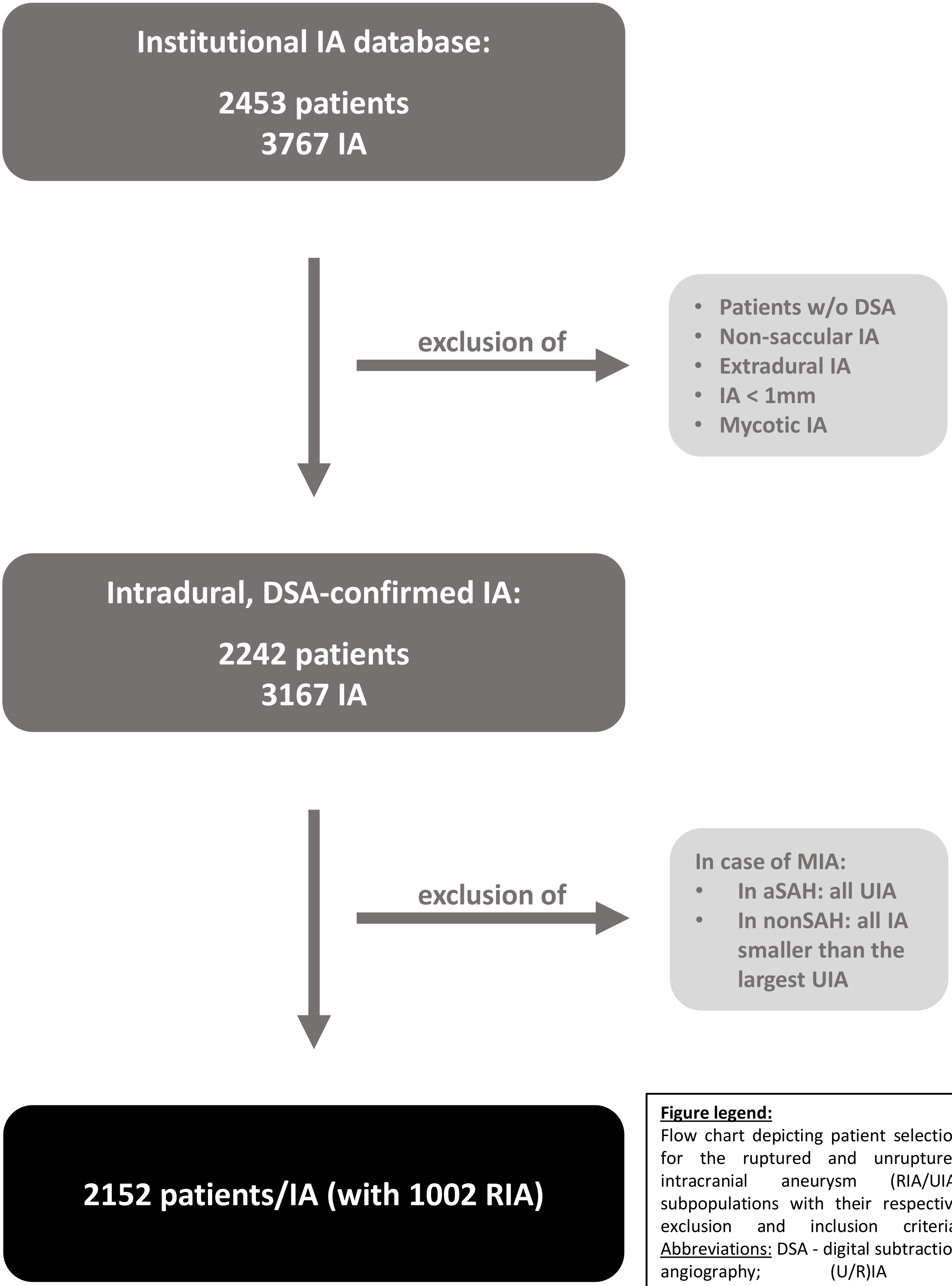

Figure legend:
Flow chart depicting patient selection for the ruptured and unruptured intracranial aneurysm (RIA/UIA) subpopulations with their respective exclusion and inclusion criteria. Abbreviations: DSA - digital subtraction angiography; (U/R)IA - unruptured/ruptured intracranial aneurysm; (a)SAH - aneurysmal subarachnoid hemorrhage.

Figure S2

A Kruskal-Wallis post hoc analysis for IA location

UIA	H	SE	Std. H	Sig.	Adj. Sig.[a]
MCA vs. ICA	-54.31	24.85	-2.19	0.029	0.173
ACA vs. ICA	70.40	29.00	2.43	0.015	0.091
ICA vs. PC	-59.35	28.84	-2.06	0.040	0.238
ACA vs. MCA	16.09	28.08	0.57	0.567	1.00
ACA vs. PC	-129.75	31.67	-4.10	<0.001	<0.001
MCA vs. PC	-113.66	27.92	-4.07	<0.001	<0.001

RIA	H	SE	Std. H	Sig.	Adj. Sig.[a]
MCA vs. ICA	-18.37	32.62	-0.56	0.573	1.000
ACA vs. ICA	122.70	29.87	4.11	<0.001	<0.001
PC vs. ICA	71.29	31.15	2.29	0.022	0.133
ACA vs. MCA	104.33	24.59	4.24	<0.001	<0.001
ACA vs. PC	-51.41	22.61	-2.27	0.023	0.138
PC vs. MCA	52.93	26.14	2.03	0.043	0.257

B Pairwise comparison of IA location

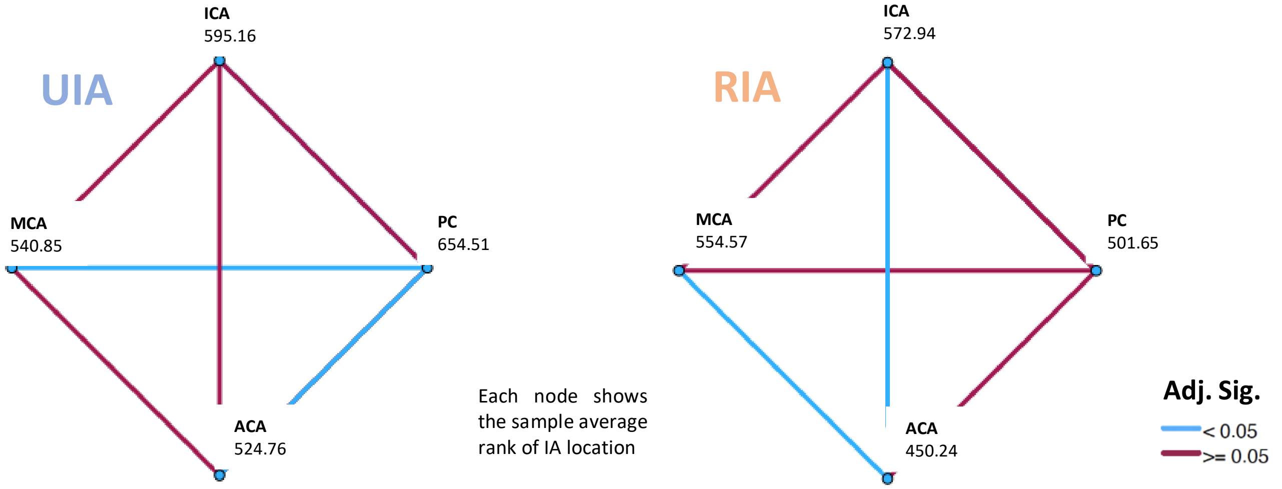

Figure legend:
(A) Tables showing the results of the Kruskal-Wallis post hoc analysis for IA location with the test statistic H, standard error (SE), the significance with a cut-off defined by $p<0.05$ and an adjusted significance for UIA (upper part, blue) and RIA (lower part, orange).
(B) Node diagram highlighting significant pairwise comparisons (blue) and non significant comparisons (purple) (left side UIA; right side RIA).
Abbreviations: ACA – anterior cerebral artery; ICA – internal carotid artery; MCA – middle cerebral artery; PC – posterior circulation; (U/R)IA – (unruptured / ruptured) intracranial aneurysm.

Table S1

The table shows the distribution of (potential) intracranial aneurysm (IA) size risk/protective factors in the subgroup of patients with unruptured IA (nonSAH) or with an aneurysmal subarachnoid hemorrhage (SAH), respectively. In nonSAH cases with multiple IA only the largest IA was included.

Abbreviations: (d/p)ACA: (distal/proximal) anterior cerebral artery; ACE: angiotensin converting enzyme; ADPKD: autosomal-dominant polycystic kidney disease; AHT: arterial hypertension; ALT: alanine transaminase; AST: aspartate transaminase; ASA: acetylsalicylic acid; AT1: angiotensin receptor 1; (t)BIL: total bilirubin; CK: creatine kinase; CRP: c-reactive protein; FIA: familial intracranial aneurysms; γ-GT: γ-glutamyltransferase; HB: hemoglobin; HCT: hematocrit; ICA: internal carotid artery; LDH: lactate dehydrogenase; MCA: middle cerebral artery; MCH: mean corpuscular/cellular hemoglobin; MCV: mean corpuscular volume; MIA: multiple intracranial aneurysms; PC: posterior circulation; PLT: platelet count; RBC: red blood cell count; TP: total protein; WBC: white blood cell count.

Parameter class / Putative RF	UIA (n=1149)	RIA (n= 1002)
**Demographic**		
Age (years)	55.1 (47-64)	54.4 (45-64)
Female	816 (71.0)	679 (67.6)
Ethnicity (non-Caucasian)	62 (5.4)	47 (4.7)
**Imaging (findings)**		
MIA	379 (33.0)	303 (30.2)
Size (mm)	8.3 (5-7)	7.4 (4-9)
(p)ACA	184 (16.0)	351 (35.0)
(d)ACA	32 (2.8)	33 (3.3)
ICA	326 (28.4)	123 (12.3)
MCA	387 (33.7)	214 (21.3)
PC	220 (19.1)	282 (28.1)
**Pre-existing medical conditions**		
Adiposity	68 (5.9)	105 (10.5)
ADPKD	30 (2.6)	9 (0.9)
AHT	703 (61.6)	689 (68.6)
Alcohol abuse	13 (1.1)	76 (7.6)
Anemia	110 (9.6)*	182 (24.6)*
Cardiac diseases	153 (13.3)	194 (19.3)
Chronic inflammation	54 (4.7)	89 (8.9)
Diabetes	115 (10.0)	94 (9.4)
Drug abuse	13 (1.1)	23 (2.3)
Dyslipidemia	252 (21.9)	132 (13.1)
FIA	56 (4.9)	12 (1.2)
Gastrointestinal diseases	111 (9.7)	162 (16.1)
Gynecologic diseases	16 (1.4)	54 (5.4)
Hepatic diseases	35 (3.0)	46 (4.6)
Hyperthyroidism	20 (1.7)	10 (1.0)
Hyperuricaemia	21 (1.8)*	28 (2.8)*
Hypothyroidism	232 (20.2)	115 (11.5)
Musculoskeletal diseases	99 (8.6)	144 (14.3)
Oncologic disease	136 (11.8)	96 (9.6)
Peripheral arterial diseases	190 (16.5)	117 (11.7)
Pulmonary diseases	94 (8.2)	119 (11.9)
Renal diseases	103 (9.0)	154 (15.3)
Current smoker	245 (21.3)	297 (29.6)
**Blood group**		
0	347 (38.5)*	283 (40.2)*
A	406 (45.1)*	314 (44.6)*
B	103 (11.4)*	77 (10.9)*
AB	45 (5.0)*	30 (4.3)*
**Blood examination**		
ALT [U/L]	26.4 (17-30)*	30.2 (16-33)*
AST [U/L]	23.6 (17-26)*	33.5 (19-32)*
(t)BIL [mg/dL]	0.50 (0.30-0.60)*	0.60 (0.40-0.70)*
Calcium [mmol/L]	2.4 (2.3-2.5)*	2.2 (2.1-2.3)*

Chloride [mmol/L]	106.1 (104-108)*	108.4 (105-111)*
CK [U/L]	96.1 (55-113)*	233.6 (61-195)*
Creatinine [mg/dL]	1.03 (0.87-1.12)*	0.94 (0.79-1.02)*
CRP [mg/dL]	2.6 (0.3-2.2)*	2.9 (0.5-3.5)*
γ-GT [U/L]	35.0 (15-38)*	46.3 (15-45)*
HB [mg/dL]	13.7 (13-15)*	13.2 (12-14)*
HCT [%]	40.8 (39-43)*	38.7 (36-42)*
LDH [U/L]	200.5 (172-222)*	221.0 (178-243)*
MCH [pg]	30.1 (29-31)*	30.5 (29-32)*
MCV [fL]	89.8 (87-93)*	89.1 (86-93)*
Phosphate [mmol/L]	3.5 (3-4)*	3.2 (3-4)*
PLT [/nL]	265.6 (218-305)*	235 (195-274)*
Potassium [mmol/L]	4.4 (4.1-4.6)*	4.0 (3.7-4.3)*
RBC [/pL]	4.6 (4.3-4.8)*	4.4 (4.0-4.7)*
Sodium [mmol/L]	141.5 (140-143)*	140.3 (138-142)*
TP [g/dL]	7.0 (6.7-7.4)*	6.5 (6.1-7.1)*
Urea [mg/dL]	15.2 (12-17)*	14.6 (10-17)*
WBC [/nL]	7.9 (6.2-9.0)*	12.7 (9.4-15.6)*
**Prescribed drugs**		
β-blocker	355 (31.6)*	154 (15.7)*
ACE-inhibitors	298 (26.5)*	170 (17.3)*
ASA	148 (13.0)*	64 (6.4)*
AT1-antagonists	168 (14.9)*	56 (5.7)*
Calcium-antagonists	218 (19.4)*	96 (9.8)*
Levothyroxine	180 (5.7)*	102 (10.2)*
Statins	214 (19.0)*	96 (9.8)*

Values are shown as number, number (%), or mean (interquartile range).
*Data could not be obtained for all patients.
